# Prognostic biomarker CCR6 and its correlation with immune infiltration in cutaneous melanoma

**DOI:** 10.3389/fonc.2023.1162406

**Published:** 2023-04-25

**Authors:** Yeltai Nurzat, Damao Dai, Julong Hu, Feiyu Zhang, Zaihuan Lin, Yang Huang, Liang Gang, Hang Ji, Xiaowen Zhang

**Affiliations:** ^1^ State Key Laboratory of Respiratory Disease, Department of Otolaryngology-Head and Neck Surgery, First Affiliated Hospital, Guangzhou Medical University, Guangzhou, Guangdong, China; ^2^ Department of Plastic and Cosmetic Surgery, Shenzhen People’s Hospital (The Second Clinical Medical College, Jinan University, The First Affiliated Hospital, Southern University of Science and Technology), Shenzhen, Guangdong, China; ^3^ Department of Operating Room, Shenzhen People’s Hospital (The Second Clinical Medical College, Jinan University, The First Affiliated Hospital, Southern University of Science and Technology), Shenzhen, Guangdong, China; ^4^ Department of Plastic Surgery, First Affiliated Hospital, Guangzhou Medical University, Guangzhou, Guangdong, China; ^5^ Department of Allergy and Clinical Immunology, First Affiliated Hospital of Guangzhou Medical University, Guangzhou, China; ^6^ Department of Cancer, First Affiliated Hospital of Guangzhou Medical University, Guangzhou, China

**Keywords:** cutaneous melanoma, CCR6, TCGA database, prognosis, biomarker

## Abstract

**Background:**

Cutaneous melanoma (CM) is an aggressive type of skin cancer. Even after standard treatment, the recurrence and malignant progression of CM were almost inevitable. The overall survival (OS) of patients with CM varied widely, making it critical for prognostic prediction. Based on the correlation between CCR6 and melanoma incidence, we aimed to investigate the prognostic role of CCR6 and its relationship with immune infiltration in CM.

**Methods:**

We obtained RNA sequencing data from The Cancer Genome Atlas (TCGA) to analyze the CM expression. Functional enrichment analyses, immune infiltration analyses, immune checkpoint analyses, and clinicopathology analyses were performed. Univariate and multivariate Cox regression analyses were used to identify independent prognostic factors. A nomogram model had been developed. Kaplan–Meier survival analysis and log-rank test were used to estimate the relationship between OS and CCR6 expression.

**Results:**

CCR6 was significantly upregulated in CM. Functional enrichment analyses revealed that CCR6 was correlated with immune response. Most immune cells and immune checkpoints were positively correlated with CCR6 expression. Kaplan–Meier analyses showed that high CCR6 expression was associated with a good outcome in CM and its subtypes. Cox regression showed that CCR6 was an independent prognostic factor in patients with CM (HR = 0.550, 95% CI = 0.332–0.912, *p*<0.05).

**Conclusions:**

CCR6 is considered to be a new prognostic biomarker for patients with CM, and our study provides a potential therapeutic target for CM treatment.

## Introduction

Cutaneous melanoma (CM) represents a skin malignancy with the highest aggressiveness and fatality, and exhibits a markedly increasing incidence worldwide over the last decade (1). Therefore, CM has greatly threatened human health globally. In recent years, the relation of molecular biomarkers to cancer survival has gained great attention in cancer occurrence. For example, autophagy-related genes, including ULK1, ATG10, and ATG16L2, possess a prognostic value in glioma cohorts and were used as the biomarkers in diagnosing glioma (2–4). Moreover, hsa-miR-196a-5p, which was involved in malignant biological behaviors, was suggested to be a poor prognostic factor of glioma (5). Furthermore, increased EVA1B and EVA1C expression in glioma is tightly correlated with the high infiltration levels of multiple immune cells as well as poor prognosis (6, 7). Thus, the wide use of biomarkers in cancer prediction and diagnosis drives considerable research efforts in the identification of biomarkers able to predict the CM. At this point, ERBB1/2/3 are suggested to serve as early prognostic markers and potential therapeutic targets in CM (8). Moreover, immune responses are currently under investigation in predictive biomarkers of CM. For example, the specific components of CM microenvironment and, in particular, the CD8+ T-cell activation, through IFN-γ gene expression signature, have been associated with immune response. Moreover, several studies have demonstrated the mechanisms through which specific genomic alterations can drive immune checkpoint resistance through the alteration of antigen-presenting mechanisms and IFN-γ production (9). Recently, in humans, it has been demonstrated that specific gut microbiota compositions can drive differential responses to immune checkpoint inhibitors (10). Nonetheless, there is a lack of convincing prognostic prediction molecules for CM at different stages. Consequently, it is difficult to provide prognostic estimations according to the patient’s condition in clinical practice, and more effective biomarkers are required. Multiple studies, including the landmark Cancer Genome Atlas Project (TCGA) for CM, have revealed common CM mutations and features that are highly characteristic of CM. As regards the identification of complex biological interactions among different pathways and their interplay with the immune system, bioinformatics has yielded promising results (11).

CCR6 encodes a G-protein-coupled receptor belonging to the β-chemokine receptor family (12, 13). It is expressed in memory T cells as well as immature dendritic cells, and macrophage inflammatory protein 3α (MIP-3α) is its ligand (14). CCR6 is tightly associated with maturation and differentiation of B cells and can also affect the migration and recruitment of T cells (12, 13). CCR6 may be associated with autoimmune disorder through regulating the GPCR and CCR5 signaling pathway (15). Moreover, CCR6 is associated with biological activities, such as chemokine receptor activity and G-protein-coupled receptor activity (16). Owing to the interaction with several immune cells, CCR6 has been found to regulate the immune microenvironment in CM. First, abnormal expression of CCR6 is recently discovered in CM, with the poor clinic prognosis rate (17). Additionally, the interaction between CCR6 and CCL20, which plays a decisive role in melanoma pathogenesis beyond chemoattraction, is considered to be important for CM development (18). The activation of CCR6 by CCL20 may be linked to the recruitment of a variety of immune cells (19–22). Based on the correlation between CCR6 and melanoma incidence, we suggest that CCR6 has great potential as an independent prognostic predictor of clinical outcomes and may be important for diagnosing and preventing melanoma.

CCR6 expression profiles within CM were analyzed using the TCGA database, and the significance in prognosis was assessed. CCR6 was found to be upregulated in CM, which predicted the positive prognostic outcome for CM cases. Furthermore, CCR6 was related to immune checkpoints and immune responses, providing new insights for individualized treatment. Consequently, CCR6 was the prognostic factor and anti-CM therapeutic target.

## Methods

### Collection of RNA sequencing data

RNA sequencing (RNA-seq) data from GTEx and TCGA databases in pan-cancer were collected using the Toil process in UCSC XENA (https://xenabrowser.net/datapage/) (23, 24). In subsequent examination, HTSeq-Count and HTSeq-FPKM data at level 3 from 529 TCGA-LGG cases (https://portal.gdc.cancer.gov/) were collected. The present work followed guidelines from the TCGA and GTEx databases.

### Differentially expressed gene identification

This work selected median *CCR6* level to be the threshold for identifying differentially expressed genes (DEGs) of high and low *CCR6* expression groups within CM samples (HTSeq-Count). DEGs were analyzed by the DESeq2 R package (1.26.0) (25).

### Functional annotation

DEG thresholds used in functional annotation included adjusted *p* < 0.05 and |logFC| > 2. In addition, Gene Ontology (GO) analysis, including biological processes (BPs), cellular components (CCs), and molecular functions (MFs), together with Kyoto Encyclopedia of Genes and Genomes (KEGG) analysis was carried out using the ClusterProfiler R package (3.14.3) (26, 27).

### Gene set enrichment analysis

The ClusterProfiler R package (3.14.3) was adopted for exploring differences in functions and pathways of both groups with diverse *CCR6* levels (28). Permutation numbers were 1,000 in all analyses. Statistical significance in enrichment analysis was deemed upon FDR *q*-value < 0.25 and *p*.adj < 0.05.

### Immune infiltration as well as immune checkpoint analysis

By using the GSVA R package (1.34.0), immune infiltration on *CCR6* was carried out *via* single-sample Gene Set Enrichment Analysis (ssGSEA) (29). According to a previous description, we enrolled 24 tumor-infiltrating immune cells (TIICs) for analysis (30). Moreover, the relation of *CCR6* to immune checkpoints, such as PD-L1, PD1, LAG3, CTLA4, TIGIT, TIM3, and CD48, was examined (31).

### Construction of a prognosis prediction model

Univariate/multivariate Cox regression was performed to evaluate whether *CCR6* was a factor to independently predict prognosis. This work included clinical factors, such as age, sex, TNM stage, and body mass index (BMI). In addition, RMS (version 6.2–0) was adopted for nomogram construction, while survival (version 3.2–10) was selected for calibration plot generation to predict the 1-, 3-, and 5-year overall survival (OS) (32). Additionally, identical factors to those in Cox regression analysis were selected. The nomogram-estimated probabilities were mapped for the graphical evaluation of the calibration plot against the real measurements. Moreover, we utilized a diagonal value to be the optimal predicting significance. Discrimination was analyzed using the concordance index (C-index) with 1,000 bootstrapping replicates (33). Additionally, the nomogram’s prediction performance was assessed by a receiver operating characteristic (ROC) curve.

### Survival analysis verification

The present work collected gene expression profiles as well as clinicopathological data from 625 CM cases in two RNA-seq datasets from TCGA (34). This was adopted as a validation set for verifying survival analysis together with *CCR6*’s prognostic significance.

### Cell proliferation and colony formation assays

Cell proliferation was evaluated with MTT (Solarbio) reagent. Cells were plated in 96-well plates (3 × 10^3^ cells per well), and the absorbance was measured at different time points, as indicated in the text. After the indicated operation, 25 μl of MTT solution (5 mg/ml) was added to each plate well. After incubation for 1 h, 100 µl of DMSO (BioFroxx) was used to dissolve the purple formazan crystals. The absorbance at 490 nm was measured using a microplate reader.

### Invasion assay

Invasion assays were performed using 24-well transwell plates with an 8.0-μm pore polycarbonate membrane insert (Corning, NY). Cells were washed twice in serum-free culture medium and re-suspended to 3 × 10^4^ cells/100 μl with serum-free medium. Resuspended cells were seeded on the upper chamber covered with Matrigel (BD Biosciences, USA). Conditioned medium (50% FBS, 500 μl) was added to the lower 24-well plates. After incubating for 48 h, respectively, were fixed with cold paraformaldehyde for 30 min. Then, cells on the upper chamber were scraped and stained with 0.1% crystal violet for 20 minutes. Five visual fields in the chamber were randomly selected and photographed using a Nikon Eclipse TS100 microscope.

### Reverse transcriptase polymerase chain reaction

Total RNA was extracted using TRIzol reagent (Tiangen Biotech, Beijing, China), followed by reversely synthesizing cDNA with the PrimeScript™ RT Reagent Kit with gDNA Eraser (TaKaRa). The reverse transcriptase polymerase chain reaction (RT-qPCR) condition is as follows: 95°C for 30 s, followed by 30 cycles at 95°C for 30 s, 60°C for 1 min, and 72°C for 30 s.

### Western blotting

Standardized equal amounts of protein were added to sodium dodecyl sulfate (SDS)-polyacrylamide gel electrophoresis for 90 min. Then, the proteins were transferred to polyvinylidene difluoride (PVDF) membranes (Millipore, Bedford, MA, USA). After blocking in 5% bovine serum albumin (BSA), PVDF was incubated with primary antibody overnight at 4°C. The primary antibodies against CCR6 (ab227036, Abcam) and GAPDH (ab8245, Abcam) were used according to the manufacturer’s guidelines. Then, the PVDF was incubated with the secondary antibodies for 1 h at room temperature. HRP-conjugated secondary antibody (AS061, Abclonal) was used according to the manufacturer.

### Statistical analysis

Statistical analysis was conducted and then a graph was generated using R language (version 3.6.3). Later, *CCR6* levels were examined using the unpaired-sample Wilcoxon rank-sum test. Cox regression was used to assess hazard ratios (HRs) together with 95% confidence intervals (CIs) for diverse clinical features and identify factors independently predicting prognosis. We adopted log-rank test and Kaplan–Meier (KM) survival analysis for predicting survival distribution. *p* < 0.05 (two-sided) indicated statistical significance.

## Results

### CCR6 levels within pan-cancers and CM

**Figure 1:**
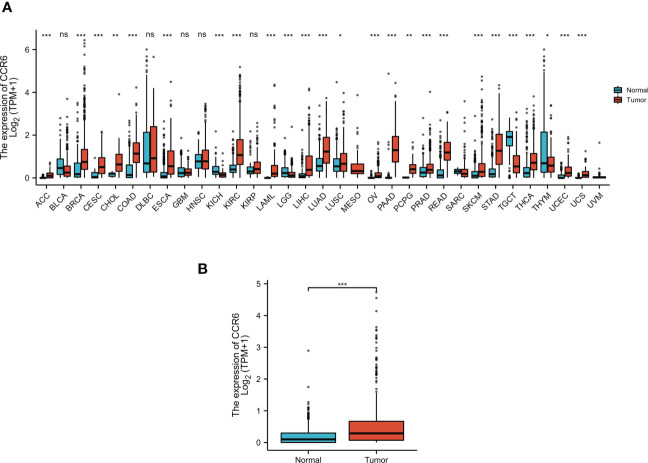
The expression pattern of CCR6 in different samples. **(A)** CCR6 expression between normal tissues and pan-cancer samples. **(B)** CCR6 expression between normal tissues and skin cutaneous melanoma (SKCM). *p < 0.05; **p < 0.01; ***p < 0.001. NS, not statistically significant.

**Figure 2:**
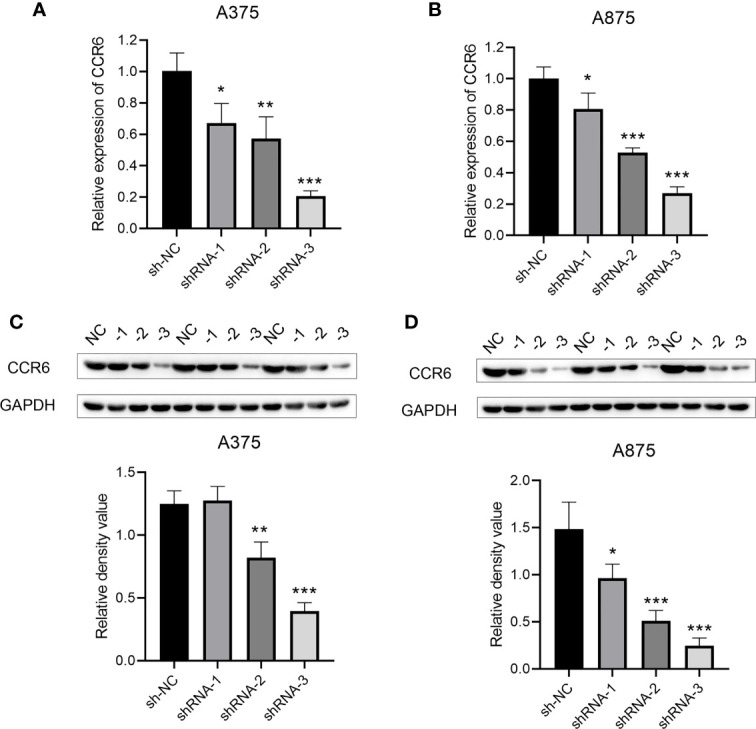
The mRNA **(A, B)** and protein level **(C, D)** of CCR6 in A375 and A873 cell lines by qRT-PCR and Western blotting analysis. *p<0.05; **p<0.01; ***p<0.001.

**Figure 3:**
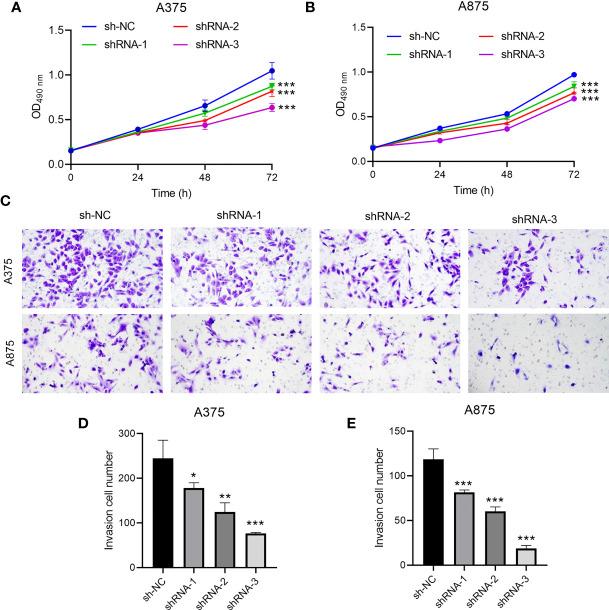
The effect of CCR6 on proliferation **(A, B)** and invasion **(C–E)** of A375 and A873 cells. *p<0.05; **p<0.01; ***p<0.001.

CCR6 levels were compared in cancer and non-carcinoma samples in GTEx and TCGA databases; as a result, CCR6 showed significant upregulation within many cancers (**Figure 1A**), such as CM (*p* < 0.05, **Figure 1B**). Next, the sh-RNA specific to CCR6 was introduced to explore the effect of CCR6 on tumorigenesis in the A375 and A875 cell lines. The qRT-PCR and Western blotting results showed that the shRNA-3 successfully knocked down the expression of CCR6 in both A375 and A875 cell lines (**Figures 2A–D**). MTT assays showed that knockdown of CCR6 by sh-RNA transfection significantly inhibited A375 cell proliferation compared with that of the control cells (sh-NC-transfected) (**Figure 3A**). MTT assays were further performed and showed that CCR6 deficiency also blocked the proliferation of A875 cells (**Figure 3B**). Moreover, the transwell assay and quantitative data also demonstrated that CCR6 knockdown significantly decreased the invasion of A375 and A875 cells (**Figures 3C–E**).

### DEG identification with CCR6 and functional enrichment analyses

**Figure 4:**
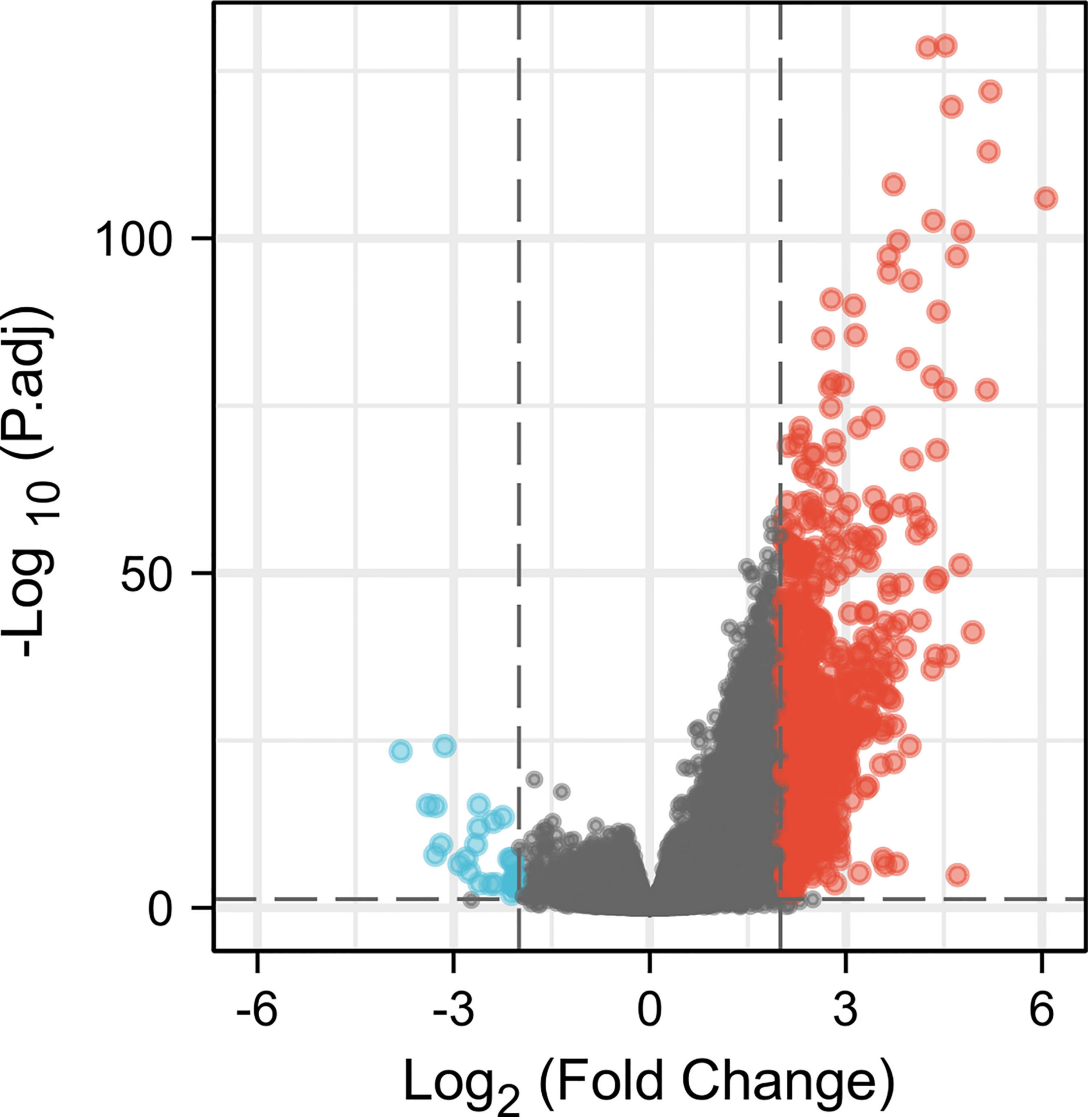
A total of 678 upregulated and 31 downregulated genes were identified as being statistically significant between CCR6 high-expression and low-expression groups.

There were altogether 709 DEGs detected from high and low CCR6 expression groups upon *p*.adj < 0.05 and |logFC| > 2 thresholds, which included 678 upregulated genes as well as 31 downregulated ones (**Figure 4**).

**Figure 5:**
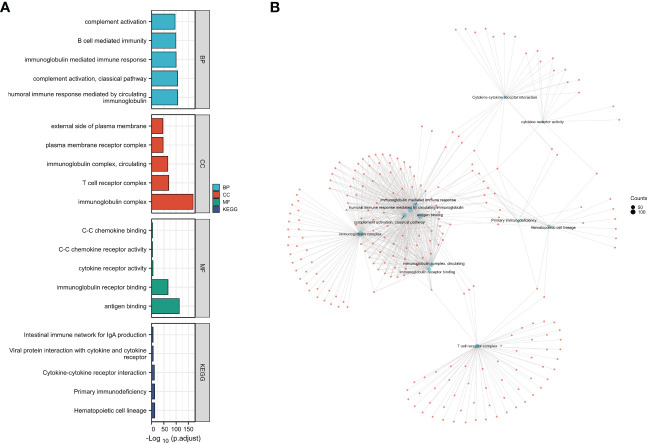
Kyoto Encyclopedia of Genes and Genomes **(KEGG)** and Gene Ontology (GO) analysis of differentially expressed genes (DEGs) in CCR6. **(A)** GO functional analysis and KEGG enrichment analysis of DEGs in CCR6. **(B)** The connection of different GO functional items; each node refers to the DEGs in GO items.

GO as well as KEGG analysis was conducted to identify important pathways that are involved in the molecular activity of CCR6. BPs included the humoral immunity regulated through complement activation, circulatory immunoglobulin, B cell-mediated immunity, and immunoglobulin-mediated immune response. CCs included immunoglobulin complex, T-cell receptor complex, external side of the plasma membrane, and circulating plasma membrane receptor complex. MFs include antigen binding, cytokine receptor activity, immunoglobulin receptor binding, C-C chemokine binding, and C-C chemokine receptor activity. KEGG included hematopoietic cell lineage, cytokine–cytokine receptor interactions, primary immunodeficiency, the intestinal immune network related to IgA generation, and viral protein interactions with cytokines and cytokine receptors (**Figure 5**).

**Figure 6:**
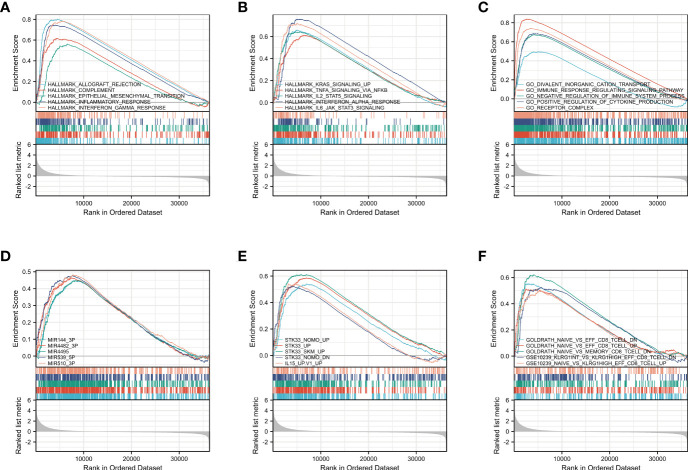
Enrichment analyses from gene set enrichment analysis (GSEA) **(A–F)**.

GSEA was carried out to identify biological activities related to CM at diverse CCR6 levels through MSigDB collection. Of significantly enriched genes, 10 hallmark categories, namely, complement, allograft rejection, inflammatory response, epithelial mesenchymal transition, KRAS pathway up, TNFA pathway *via* NF-κB, IL2-STAT5 pathway, IFN-α response, IFN-γ response, and IL-6-JAK-STAT3 pathway, were markedly differentially enriched into the CCR6 upregulation phenotype (**Figures 6A, B**). Five GO terms, namely, divalent inorganic cation transport, immune response-regulating signaling pathways, positive regulation of cytokine production, receptor complexes, and negative regulation of immune system process, were markedly differentially enriched into the CCR6 upregulation phenotype (**Figure 6C**). Furthermore, five MOTIF terms, namely, MIR-144-3P, MIR4482-3P, MIR4495, MIR539-5P, and MIR510-3P, were markedly differentially enriched into the CCR6 upregulation phenotype (**Figure 6D**); five oncogenic signature terms, namely, STK33-NOMO-UP, STK33-UP, STK33-SKM-UP, STK33-NOMO-DN, and IL15-UP, were markedly differentially enriched into the CCR6 upregulation phenotype (**Figure 6E**). Five immunological signature terms associated with CD8+ T cells showed significantly differential enrichment in the CCR6 upregulation phenotype (**Figure 6F**).

Based on the above findings, CCR6 might be important for the immune response together with the tumor microenvironment (TME), and these were of great importance for CM cases.

### Immune infiltration analysis

**Figure 7:**
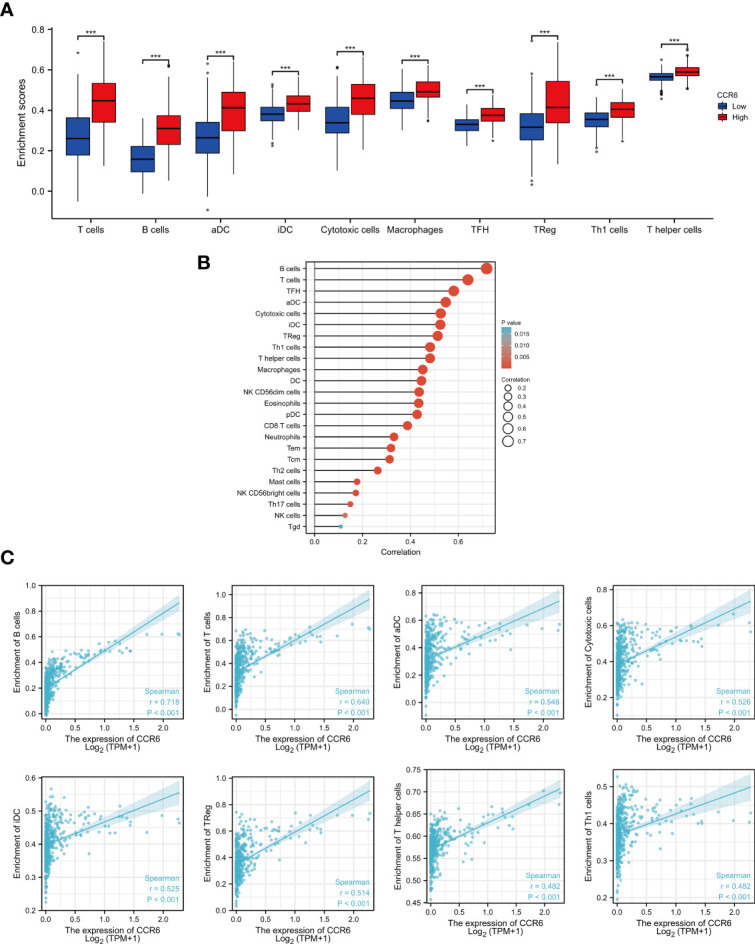
Relation of CCR6 level to immune infiltration within cutaneous melanoma (CM). **(A)** Infiltration degrees of 10 immune cell subtypes between CCR6 up- and downregulation groups. **(B)** Association of CCR6 level with different immune cells. **(C)** Association of CCR6 level with immune infiltration degrees.

Tumor immune infiltration plays a crucial role in predicting the OS of CM. According to percentages of 10 immune cell subtypes within the diverse CCR6 level groups, B cells (*p* < 0.001), T cells (*p* < 0.001), iDC (*p* < 0.001), aDC (*p* < 0.001), macrophages (*p* < 0.001), cytotoxic cells (*p* < 0.001), Treg (*p* < 0.001), TFH (*p* < 0.001), Th1 cells (*p* < 0.001), and T helper cells (*p* < 0.001) were remarkably elevated in CCR6 upregulation groups (**Figure 7A**). Additionally, these indicated positive correlations of CCR6 level with the infiltration degrees of different immune cells. Among them, B and T cells were markedly related to CCR6 level within CM (**Figure 7B**). Specifically, the infiltration of B cells (*r* = 0.718, *p* < 0.01), T cells (*r* = 0.640, *p* < 0.01), aDC (*r* = 0.548, *p* < 0.01), cytotoxic cells (*r* = 0.526, *p* < 0.01), iDC (*r* = 0.525, *p* < 0.01), Tregs (*r* = 0.514, *p* < 0.01), T helper cells (*r* = 0.482, *p* < 0.01), and Th1 cells (*r* = 0.482, *p* < 0.01) was closely related to CCR6 expression in CM (**Figure 7C**).

**Figure 8:**
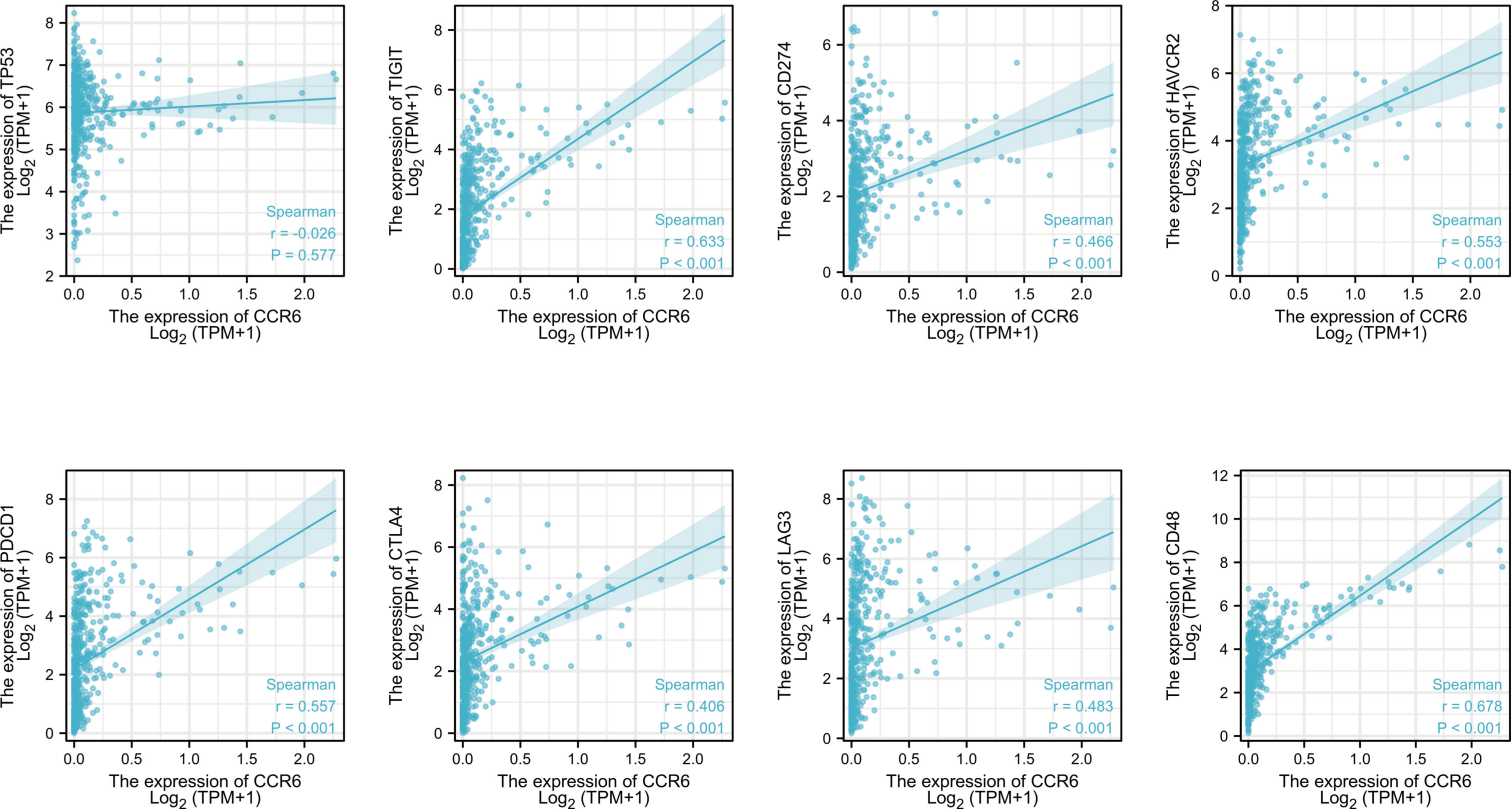
Association between CCR6 expression and immune checkpoints.

Additionally, the relation of CCR6 level to immune checkpoints, such as TP53, PD1, PD-L1, CTLA4, LAG-3, TIM3, TIGIT, and CD48, was examined (**Figure 6**). PD1 (*r* = 0.557, *p*<0.001), PD-L1 (*r* = 0.466, *p*<0.001), CTLA4 (*r* = 0.406, *p*<0.001), LAG-3 (*r* = 0.483, *p*<0.001), TIM3 (*r* = 0.553, *p*<0.001), TIGIT (*r* = 0.633, *p*<0.001), and CD48 (*r* = 0.678, *p*<0.001) levels showed a positive relation to CCR6 expression (**Figure 8**).

### Association of CCR6 level with clinical features

**Figure 9:**
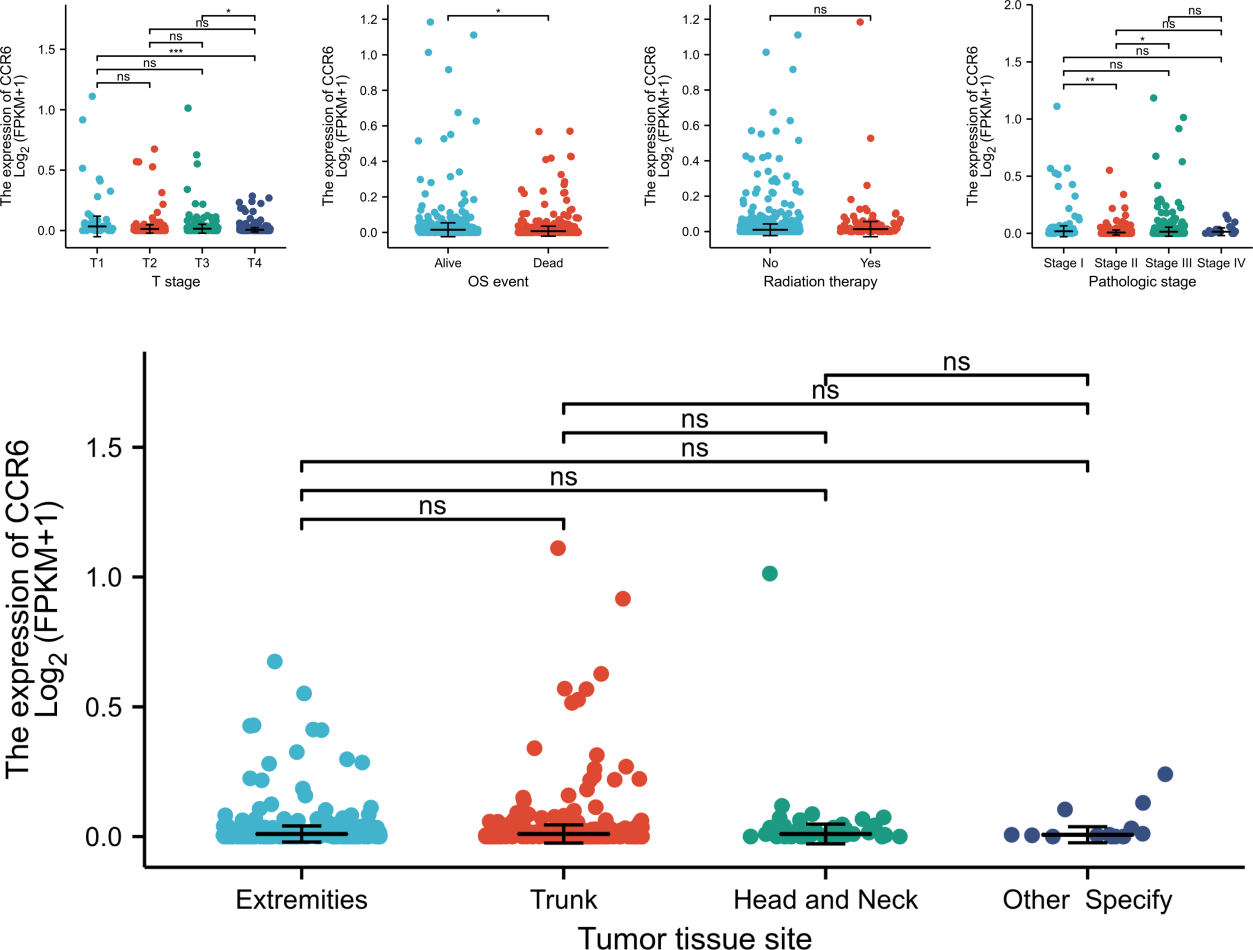
Association between CCR6 expression and clinical features. NS, not statistically significant.

To investigate the relation of CCR6 level to CM clinical features, we compared CCR6 expression against diverse clinical features (**Figure 9**). In accordance with our results, as a result, CCR6 level may only be related to late tumor stage (III to IV) and OS rate (**Table 1**).

**Table 1:** Association between CCR6 expression and clinicopathologic features in CM.

Characteristics	Total (*N*)	Univariate analysis		Multivariate analysis	
		Hazard ratio (95% CI)	*p*-value	Hazard ratio (95% CI)	*p*-value
T stage	284				
T1	41	Reference			
T3	90	2.053 (1.133–3.720)	**0.018**	1.732 (0.868–3.457)	0.119
T4	153	3.621 (2.016–6.505)	**<0.001**	3.284 (1.652–6.526)	**<0.001**
N stage	402				
N0	224	Reference			
N1	73	1.497 (1.014–2.210)	**0.043**	2.035 (1.251–3.312)	**0.004**
N2	49	1.534 (0.972–2.419)	0.066	2.655 (1.492–4.724)	**<0.001**
N3	56	2.731 (1.769–4.215)	**<0.001**	4.525 (2.472–8.284)	**<0.001**
M stage	430				
M0	406	Reference			
M1	24	1.897 (1.029–3.496)	**0.040**	2.129 (0.831–5.451)	0.115
Age	456				
≤60	246	Reference			
>60	210	1.656 (1.251–2.192)	**<0.001**	1.312 (0.890–1.935)	0.171
CCR6	456				
Low	227	Reference			
High	229	0.590 (0.450–0.773)	**<0.001**	0.654 (0.449–0.952)	**0.027**

Bold value refers to the data which is statistically significant.

### Relation of CCR6 level to prognostic outcome

**Figure 10:**
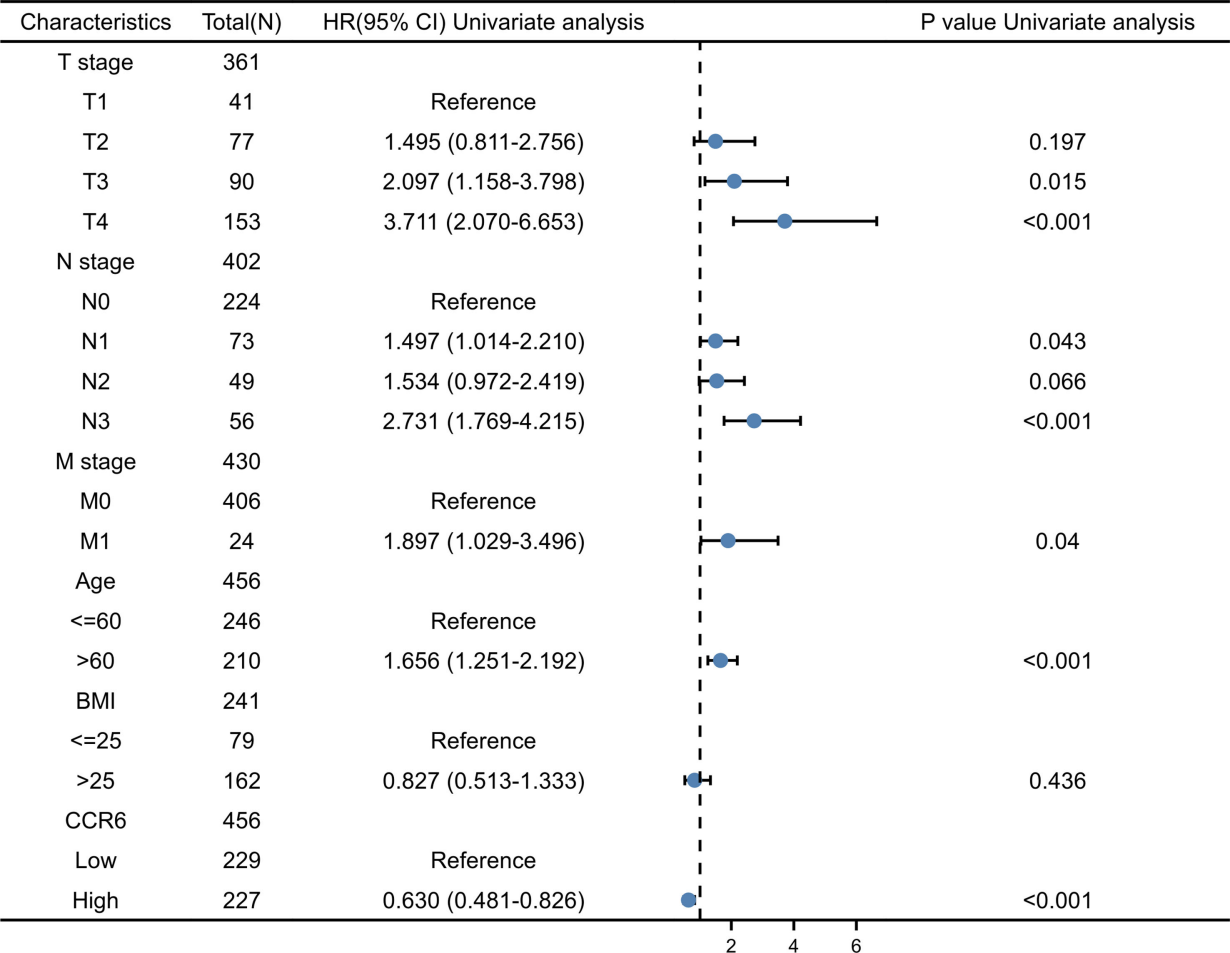
Univariate Cox analysis of CCR6 and other clinicopathological variables.

**Figure 11:**
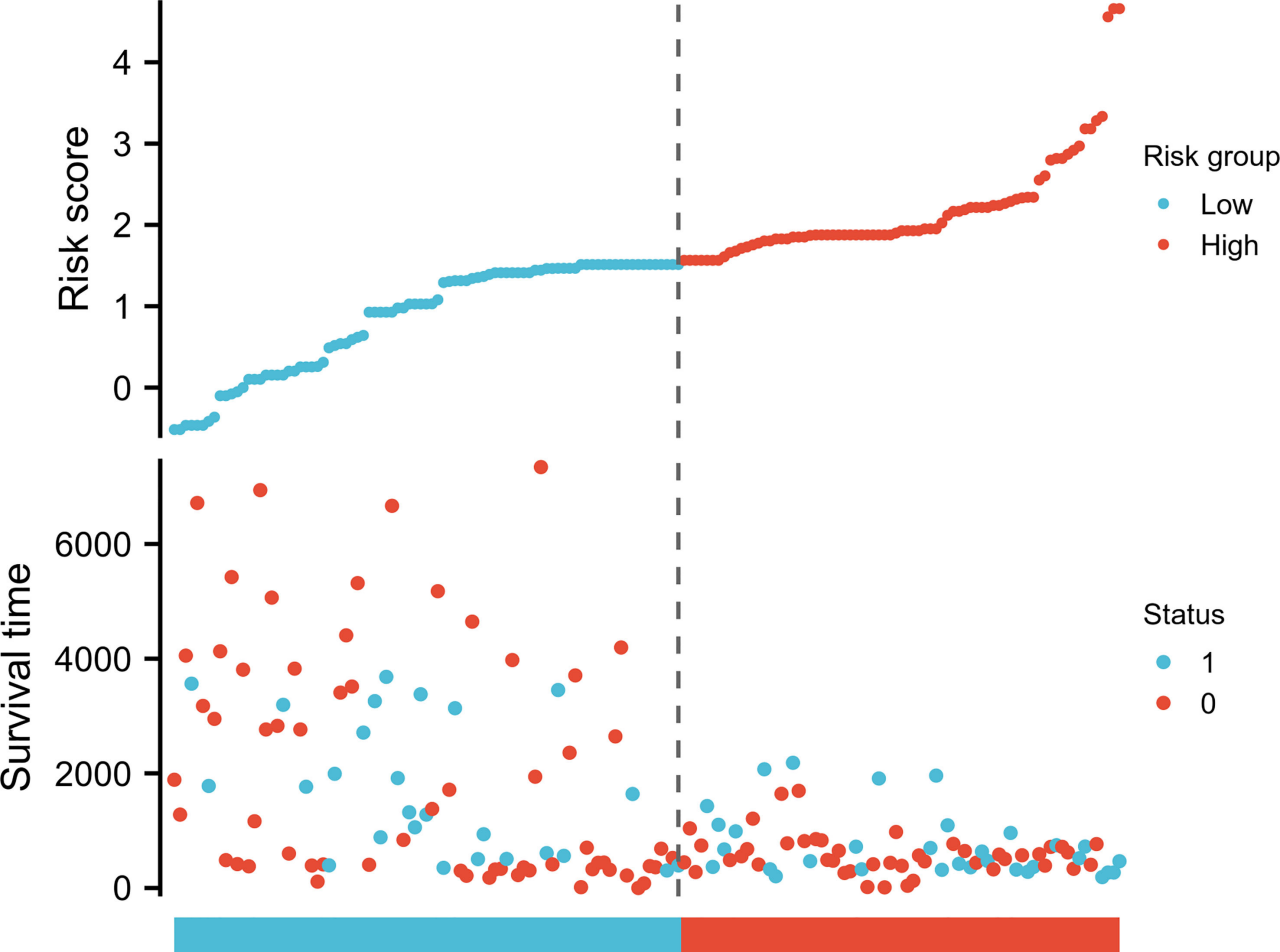
CCR6 expression, risk score, and survival time distribution.

**Figure 12:**
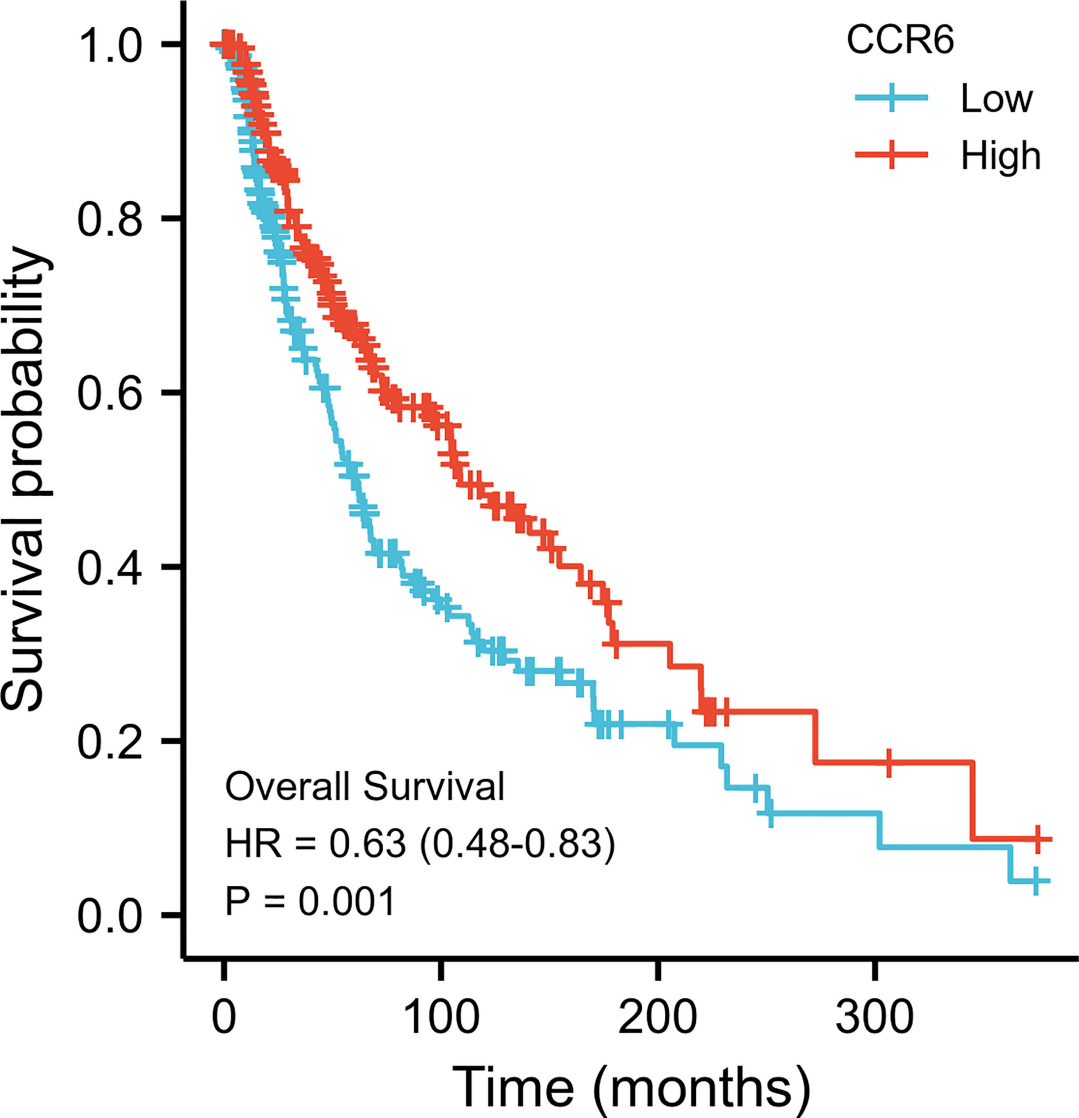
Kaplan–Meier survival analyses of CM with different CCR6 expression levels.

Possible predicting factors were examined using Cox regression, such as age, T/N/M stage, BMI, and CCR6 expression level. Upon univariate regression, age, T stage, N stage, and CCR6 expression showed marked relation to OS (*p* < 0.001). The above risk factors were later enrolled into the univariate Cox analysis (**Figure 10**). Based on our findings, CCR6 expression independently predicted patient prognosis (HR = 0.630, 95% CI = 0.481–0.826, *p* < 0.001). We later examined the relation of risk score and CCR6 expression to survival time (**Figure 11**). Kaplan–Meier (KM) curve analysis revealed the relation of CCR6 level to OS in CM cases (**Figure 12**). Cases showing CCR6 upregulation had a markedly prognostic outcome to CCR6 downregulation (HR = 0.63, 95% CI = 0.48–0.83, *p* = 0.001).

**Figure 13:**
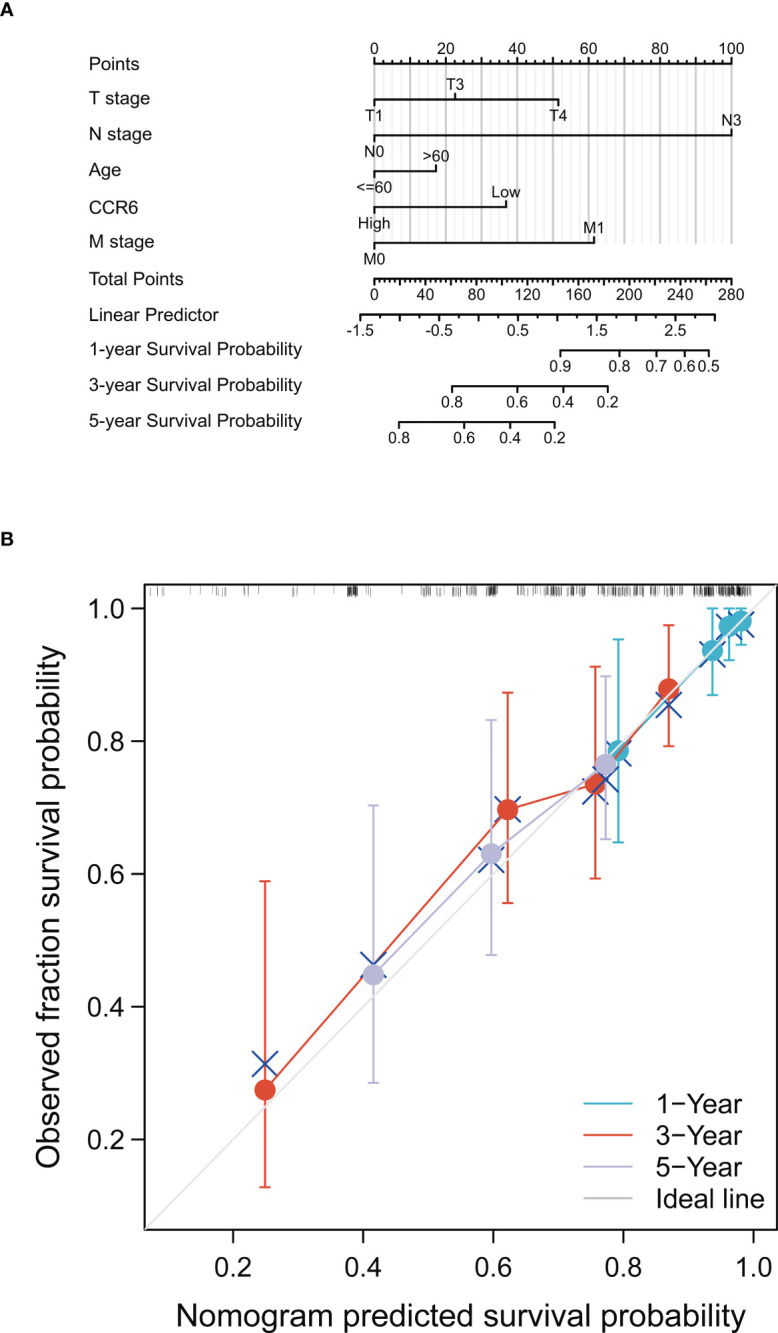
Prognostic prediction model of CCR6 in SKCM. **(A)** Nomogram for 1-year, 3-year, and 5-year OS of SKCM patients. **(B)** Calibration plots for 1-year, 3-year, and 5-year OS prediction.

This work included clinical characteristics in the disease-free survival nomogram model (**Figure 13A**), while the multivariate Cox regression HR index for CCR6 expression was 0.550 (95% CI = 0.332–0.912, *p* < 0.05). Calibration plot-estimated probabilities conformed to our measurements (**Figure 13B**).

## Discussion

CM is an aggressive type of skin cancer and is estimated as the fourth leading cause of cancer-associated mortality globally (35). Patients with primary tumors usually have an increased 5-year survival (36). Consequently, it is of great importance to detect CM early; in this case, identifying early prognostic biomarkers is critically essential in CM cases. Bioinformatic analysis is widely used to identify biomarkers of CM (37). Thus, it is necessary to identify the prognostic factors for treating patients as early as possible.

Several studies have demonstrated the interaction between tumor cells and immune responses, while immune components within melanoma samples are adopted for evaluating whether therapeutics are important to treat and predict the prognosis of CM (38). CCR6 is upregulated within lymph nodes, lung mucosa, and intestinal mucosa in patients with epithelial tumors and melanoma (16, 39–42). However, few studies have investigated whether CCR6 can be used for predicting CM prognosis. These findings showed that CCR6 level exhibited a marked relation to OS as well as immune cell infiltration of CM cases.

In the current study, CCR6 expression showed marked upregulation in many cancers, such as CM. Subsequently, we examined CCR6 expression and demonstrated that CCR6 is associated with the immune response. Following further study on the TME, immune cells had an important and complicated effect on cancer development (43–46). According to enrichment analysis, this work examined immune infiltration levels using GSEA. As a result, CCR6 expression was markedly positively related to many immune cell levels, which showed great CCR6 infiltration in the CM. Our results were consistent with an excess immune response, which indicated that the abnormal tumor immune microenvironment (TIME) reduced survival of such cases (47, 48). Among the candidates, B and T cells (*p* < 0.001) were most significantly related to CCR6 level, while their infiltration degrees were related to prognostic outcome. The increased B- and T-cell infiltration levels with high CCR6 expression in CM suggested that the change from an anti-cancer status to an immunosuppressive status triggered the TIME because of B- and T-cell composition ratios within the TIME in CM tumor samples, thereby showing indirect regulation on immune monitoring as well as the impact on cancer development (49).

Additionally, CCR6 level was positively related to immune checkpoints such as TP53, PD1, PD-L1, CTLA4, LAG-3, TIM3, TIGIT, and CD48. CCR6 might affect tumor immunology, which was the possible immunotherapeutic target but not merely the prognostic marker. Though CCR6 expression was similar in different stages of CM, it was significantly different in the T1 vs. T4 subset of WHO and OS events. This finding indicates that CCR6 has potential as a positive prognostic predictor.

The prognostic value of CCR6 among CM cases was also analyzed. According to Cox regression, CCR6 independently predicted CM prognosis, apart from conventional risk factors, such as age or TNM stage. Based on KM analysis, CCR6 was related to OS. CCR6 upregulation was associated with outcomes in CM across TNM stages. The CGGA database was adopted for verifying Cox regression and survival analysis. Furthermore, this work built a nomogram prognosis model constructed by incorporating CCR6 expression for predicting 1-, 3-, and 5-year OS for CM patients. HR index was 0.550 (95% CI = 0.332–0.912, *p*<0.05). Calibration plots indicated that our nomogram performed well in prediction. As a result, the as-constructed model offered the novel perspective to predict outcomes and assess individual prognosis of CM cases. Nonetheless, certain limitations should be noted in our study. Clinical tissues were enrolled for verification. Regulatory mechanisms as well as pathways associated with CCR6 should be investigated.

Another issue that needs further research is the role of CCR6 in inflammation during the development of TME of melanoma. The inflammatory cells in the progression of melanin and other tumors, and our enrichment analysis results implied that another possible function of CCR6 in melanoma cancer is the activation of inflammation. This possibility is further supported by the activation of immune cells in the melanoma, which is believed to be supplemented by signals from CCR6-activated immune cells. Thus, we will continue to explore the relationship between CCR6 and inflammatory cell infiltration in a subsequent work.

In this study, the risk value calculated after the establishment of the model is a reliable independent prognostic index. Compared with the conventional prognostic indexes such as age, sex, TNM stage, and BMI, the risk score created by the CCR6 gene expression can better predict the survival of patients, which also confirms that the gene-based expression signal can accurately predict the prognosis of patients with melanoma. However, this study also has some limitations. Firstly, the clinical information in the TCGA database is incomplete, especially the lack of tumor stage-related information. Secondly, the prognostic prediction model constructed in this study is based on retrospective data, and no prospective clinical studies have been carried out to verify the model. Hu et al. proposed a prognostic signature consisting of a total of 80 melanoma tissue samples that were collected from patients to predict the prognosis of patients with melanoma. Moreover, the tumor grade was detected by pathological sectioning in their study (50). Since our prognostic model is based on immune-related genes, the immune behavior of tumors is closely related to tumor stages; some information on melanoma stages may be needed to evaluate the relationship between CCR6 and immunity. More specific forms of interaction between the two may need to be further verified and studied in other databases. However, at this stage, we still believe that the CCR6 proposed in this research has the capacity to accurately predict the patients’ prognosis with melanoma and provide a new direction for new treatment strategies.

It is obscure that CCL20 promotes CM development and is considered as a negative prognostic factor in CM in many studies (18, 20, 51). Indeed, we have aimed to demonstrate whether several cytokines, including CCL20, accelerate CM development through binding the CCR6 and activating the downstream signaling in another work. To confirm this, we knocked down CCR6 expression in melanoma cells and examined whether the effect of CCL20 on CM development was attenuated or eliminated. However, this work needs further improvement, and we hope that researchers will evaluate it and give valuable advice.

## Conclusion

To sum up, CCR6 showed high expression within CM, which predicted a good prognostic outcome. This work demonstrates the novel viewpoint that CCR6 may be a potential factor that can be used to predict CM prognosis and treat CM cases. Further studies are needed to analyze the molecular mechanisms and the underlying signaling pathway of CCR6 in CM.

## Data availability statement

Publicly available datasets were analyzed in this study. This data can be found here: https://xenabrowser.net/datapage/and https://portal.gdc.cancer.gov/.

## Ethics statement

Ethics approval or specific consent procedures were not required for this study. All the data are from the public database.

## Author contributions

YN and DD carried out the experiment and wrote the manuscript with support from XZ. HJ helped supervise the project and conceived the original idea. FZ carried out manuscript writing and revision and performed the experiment. LG provided melanoma clinical samples. All authors discussed the results and contributed to the final manuscript. All authors contributed to the article and approved the submitted version.
